# Dexmedetomidine Restores Autophagic Flux, Modulates Associated microRNAs and the Cholinergic Anti-inflammatory Pathway upon LPS-Treatment in Rats

**DOI:** 10.1007/s11481-021-10003-w

**Published:** 2021-08-06

**Authors:** Widuri Kho, Clarissa von Haefen, Nadine Paeschke, Fatme Nasser, Stefanie Endesfelder, Marco Sifringer, Adrián González-López, Nadine Lanzke, Claudia D. Spies

**Affiliations:** 1 Department of Anesthesiology and Operative Intensive Care Medicine, Charité - Universitätsmedizin Berlin, Berlin, Germany; 2 Department of Neonatology, Charité - Universitätsmedizin Berlin, Berlin, Germany; 3 CIBER-Enfermedades Respiratorias, Instituto de Salud Carlos III, Madrid, Spain

**Keywords:** Dexmedetomidine, Brain, Spleen, Autophagy, microRNA, Cholinergic anti-inflammatory pathway

## Abstract

**Abstract:**

Infections and perioperative stress can lead to neuroinflammation, which in turn is linked to cognitive impairments such as postoperative delirium or postoperative cognitive dysfunctions. The α2-adrenoceptor agonist dexmedetomidine (DEX) prevents cognitive impairments and has organo-protective and anti-inflammatory properties. Macroautophagy (autophagy) regulates many biological processes, but its role in DEX-mediated anti-inflammation and the underlying mechanism of DEX remains largely unclear. We were interested how a pretreatment with DEX protects against lipopolysaccharide (LPS)-induced inflammation in adult male Wistar rats. We used Western blot and activity assays to study how DEX modulated autophagy- and apoptosis-associated proteins as well as molecules of the cholinergic anti-inflammatory pathway, and qPCR to analyse the expression of autophagy and inflammation-associated microRNAs (miRNA) in the spleen, cortex and hippocampus at different time points (6 h, 24 h, 7 d). We showed that a DEX pretreatment prevents LPS-induced impairments in autophagic flux and attenuates the LPS-induced increase in the apoptosis-associated protein cleaved poly(ADP-ribose)-polymerase (PARP) in the spleen. Both, DEX and LPS altered miRNA expression and molecules of the cholinergic anti-inflammatory pathway in the spleen and brain. While only a certain set of miRNAs was up- and/or downregulated by LPS in each tissue, which was prevented or attenuated by a DEX pretreatment in the spleen and hippocampus, all miRNAs were up- and/or downregulated by DEX itself – independent of whether or not they were altered by LPS. Our results indicate that the organo-protective effect of DEX may be mediated by autophagy, possibly by acting on associated miRNAs, and the cholinergic anti-inflammatory pathway.

**Graphical abstract:**

**Preventive effects of DEX on LPS-induced inflammation**. DEX restores the LPS-induced impairments in autophagic flux, attenuates PARP cleavage and alters molecules of the cholinergic system in the spleen. Furthermore, DEX alters and prevents LPS-induced miRNA expression changes in the spleen and brain along with LPS.

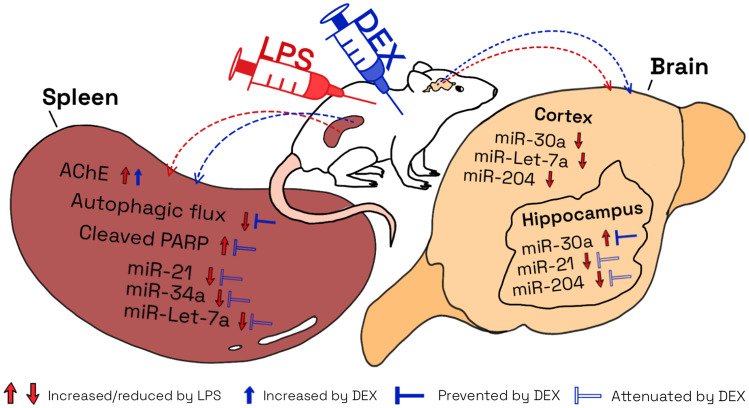

## Introduction

Cognitive impairments, such as postoperative delirium (POD) or postoperative cognitive dysfunctions (POCD) are common consequences of surgery/anesthesia and are linked to perioperative stress-induced neuroinflammation (Ntalouka et al. 2018). The elderly population is particularly affected by these pathologies, showing a prevalence of up to 53% (Saxena and Maze 2018). Due to the increase in life expectancy and surgery rates, POD/POCD are becoming a significant burden for the public health system (Ntalouka et al. 2018). Thus, controlling the immune response is a crucial step to limit neuroinflammation and thereby the risk for developing cognitive dysfunctions.

Dexmedetomidine (DEX), a α2-adrenoceptor (α2-AR) agonist, is a promising candidate to prevent cognitive impairments, as has been shown in experimental (Endesfelder et al. 2017; Paeschke et al. 2017) and clinical studies (Jakob et al. 2012; Turunen et al. 2015). In experimental studies, DEX was able to reduce the inflammatory response within the brain (Endesfelder et al. 2017; Paeschke et al. 2017) and other organs (Qiao et al. 2009; Zhao et al. 2020), but the underlying mechanism is still not fully understood. Recent studies indicate that the anti-inflammatory properties of DEX might be mediated via autophagy and the cholinergic anti-inflammatory pathway (Qiao et al. 2009; Zhao et al. 2020).

Autophagy, the internal waste reduction and recycling system of cells, is an essential cellular process for maintaining cell homeostasis and adaption to physiological stress. It is particularly important for neuron survival and the regulation of immune response (Deretic et al. 2013; Nixon 2013; Puleston and Simon 2014). Neurons, as postmitotic cells, rely heavily on autophagy to dilute the accumulated cellular waste otherwise controlled by cell division (Nixon 2013), while in the immune system, autophagy controls inflammation in a number of different ways. It balances the secretion of pro- and anti-inflammatory cytokines in macrophages, dendritic cells and microglia (Harris 2011; Plaza-Zabala et al. 2017), contributes to antigen presentation (Puleston and Simon 2014) and regulates Th17 polarization (Deretic et al. 2013) as well as B- and T-cell development and survival (Kuballa et al. 2012). Disruption of neuronal and glial autophagy are associated with a wide range of neurological diseases, such as amyotrophic lateral sclerosis, Parkinson’s or Alzheimer’s disease (Nixon 2013; Plaza-Zabala et al. 2017) and due to its central role within the immune system, disruptions of immune cell autophagy also have detrimental outcomes (Deretic et al. 2013).

Autophagy, as a highly dynamic process, undergoes complex regulatory mechanisms, including the modulation by microRNAs (miRNAs) (Feng et al. 2015). These small non-coding single stranded RNAs regulate gene expression on a post-transcriptional level and have been studied in depth regarding their regulatory role in autophagy, apoptosis and immune cell development and function (Stachurska et al. 2014; Su et al. 2016).

Furthermore, the cholinergic anti-inflammatory pathway, a communication route between the central nervous system (CNS) and the immune system, also greatly regulates the immune system by restricting the pro-inflammatory response, and the spleen is greatly involved in this pathway (Pavlov et al. 2003). It has recently been shown that the cholinergic anti-inflammatory pathway is also associated with autophagy (Shao et al. 2017) and might be involved in the DEX mediated effects (Zhu et al. 2016).

Therefore, to understand the working mechanism of DEX during inflammation, we used lipopolysaccharide (LPS) to generate an inflammation model in adult male Wistar rats. We previously showed that both, peripheral surgery combined with LPS as well as an LPS injection alone, trigger a pro-inflammatory immune response and impair autophagy within the rat brain (Kalb et al. 2013; Paeschke et al. 2017; von Haefen et al. 2018). In this study, we have now investigated the effect of DEX on the immune system, autophagy and associated miRNAs, and the cholinergic anti-inflammatory pathway in the spleen and brain.

## Materials and Methods

### Animal Model

A total of 94 male Wistar rats (250 – 300 g) aged three months were obtained from Charité-Universitätsmedizin Berlin (Germany) and used in this study. All rats were housed at room temperature (22 ± 2 °C) under a standard 12-12 h light-dark cycle and constant humidity (40 – 70%). Food and water were available *ad libitum*. To induce a systemic inflammation, the rats were injected intraperitoneally (i.p.) with LPS (1 mg/kg body weight) in the presence or absence of a prior i.p. injection of DEX (dexdor®, Orion Pharma, Espoo, Finland; 5 µg/kg body weight) or control vehicle NaCl (0.9%), which was administered 10 min prior to the LPS injection. The animals were randomized into different experimental groups: a) NaCl, b) LPS, c) DEX and d) DEX+LPS. Animals were sacrificed at three different time points: 6 h, 24 h and 7 d. Prior to any injections, animals underwent a short isoflurane-oxygen anesthesia, to minimize the influence of the animals’ individual stress responses on the injections and handling. All animal experiments were approved and performed in accordance with the guidelines of the Charité - Universitätsmedizin Berlin, Germany and the national ethic principles (registration no. G 0145/13, 1 July 2013).

### Tissue Preparation

Animals were sacrificed under deep isoflurane-oxygen narcosis by transcardial perfusion with PBS (pH 7.4) at the specified time points. Spleens were collected and immediately snap frozen. Brains were harvested and immediately microdissected into the cortical and hippocampal tissue and then snap frozen in liquid nitrogen. Samples were kept at -80 °C for further biochemical analysis.

### Immunoblotting

Tissue samples (80 – 100 mg) were homogenized in a standard RIPA buffer (Sigma Aldrich, St. Louis, MO, USA, #R0278) containing cOmplete™, mini, EDTA-free (Roche, Mannheim, Germany) and PhosSTOP™ (Roche, Mannheim, Germany) using a tissue homogenizer (FastPrep®-24, MP Biomedicals, Irvine, CA, USA). The homogenates were fractionated by centrifugation at 3,000 g and 4 °C for 10 min, followed by a centrifugation at 17,000 g and 4 °C for 20 min. The remaining cytosolic fraction was used for analyses. Protein concentrations were measured by using the bicinchoninic acid (BCA) assay from Thermo SCIENTIFIC (Rockford, IL, USA) following the manufacturer’s instructions.

For Western blot analyses 20 µg of protein from each sample were separated by a 10% polyacrylamide gel electrophoresis and transferred to nitrocellulose membranes (0.2 μm pore, Bio-Rad, Munich, Germany). Prior to primary antibody incubation overnight, membranes were blocked with 5% low fat milk solution (Carl ROTH, Karlsruhe, Germany) for 1 h at room temperature. The primary antibodies used in this study are shown in Table 1. All primary antibodies were dissolved in 1x PBST with 1% BSA. For normalization and protein loading control, membranes were incubated with monoclonal mouse anti-β-Actin-Peroxidase or monoclonal rabbit anti-β-Tubulin antibodies at concentrations of 1:50,000 and 1:1,000 in 1x PBST respectively. Horseradish peroxidase-conjugated secondary antibodies (Southern Biotechnology Associates, Birmingham, AL, USA, anti-rabbit, cat. no. 4020–05) were diluted 1:25,000 in 1x PBST. Chemiluminescence signals were detected with Immobilon Forte Western HRP substrate (Merck, Darmstadt, Germany) at a FUSION Solo S Vilber Lourmat detection system (Vilber Lourmat, Eberhardzell, Germany), using the Fusion® Software (Vilber Lourmat, Eberhardzell, Germany).

**Table 1:** Primary Antibodies

Antibody	Dilution	Company	Catalogue Number
rabbit anti-Atg5	1:1,000	Cell Signaling, Beverly, MA, USA	#12994
rabbit anti-SQSTM1/p62	1:1,000	Cell Signaling, Beverly, MA, USA	#5114
rabbit anti-LC3A/B	1:1,000	Cell Signaling, Beverly, MA, USA	#4108
rabbit anti-Bcl-2	1:1,000	Cell Signaling, Beverly, MA, USA	#2870
rabbit anti-Cytochrome C	1:1,000	Cell Signaling, Beverly, MA, USA	#11940
rabbit anti-PARP	1:1,000	Cell Signaling, Beverly, MA, USA	#9542
rabbit anti-nicotinic Acetylcholine R alpha 7/ CHRNA7	1:1,000	Novus Biologicals, Abingdon, United Kingdom	NBP1-79948
mouse anti-β-Actin-Peroxidase	1:50,000	Sigma Aldrich, St. Louis, MO, USA	#A3854
rabbit anti-β-Tubulin	1:1,000	Cell Signaling, Beverly, MA, USA	#2128

### Acetylcholine/Acetylcholinesterase Assay

The Amplex® Red Acetylcholine/Acetylcholinesterase Assay Kit (Invitrogen®, Karlsruhe, Germany, A1227) was used to measure the ACh expression and AChE activity within the tissues. Again, the cytosolic fractions of the tissue homogenates were used for this assay. In brief, for measurements a standard curve of ACh and AChE respectively was prepared, and the tissue probes were diluted with 1x reaction buffer appropriately. Afterwards, standard curve probes and diluted tissue probes were pipetted into a black 96-well plate (BRAND® microplate BRANDplates®, Wertheim, Germany, 781668) and a working solution containing 400 μM Amplex Red reagent, 2 U/mL HRP, 0.2 U/mL choline oxidase and 1 U/mL acetylcholinesterase for ACh analyses or 400 μM Amplex Red reagent, 2 U/mL HRP, 0.2 U/mL choline oxidase and 100 μM acetylcholine for AChE analyses were prepared and added to standard curve probes and tissue probes in a 1:1 ratio. All probes were measured twice to avoid measuring errors and a solution containing only 1x reaction buffer was used as negative control. The probes were measured with a fluorescence microplate reader (Tecan Infinite® M200, Zurich, Switzerland) with an excitation at 560 nm and emission detection at 590 nm.

### Semiquantitative Real-Time PCR and miRNA expression analysis

The total RNA containing miRNA was isolated from snap frozen cortical, hippocampal and splenic tissue by acidic phenol/chloroform extraction (peqGOLD RNAPure™ System, VWR, Darmstadt, Germany) according to the manufacturer’s instruction.

For analyses on miRNA level, the method published by Balcells et al. (Balcells et al. 2011) was used, which is based on polyadenylating the miRNA prior to the reverse transcription with a special RT-primer. Therefore, 500 ng total RNA containing miRNA was polyadenylated by using a 0.75 U Poly-A-Polymerase (New England Biolabs, Frankfurt am Main, Germany) and then reversely transcribed with 1 µM RT-primer (5´-CAGGTCCAGTTTTTTTTTTTTTTT-3´), 0.1 mM dNTP mix, 1 mM ATP, 80 U M-MLV reverse transcriptase (Promega, Mannheim, Germany) and 1 µL of 10x poly(A)polymerase buffer (New England Biolabs, Frankfurt am Main, Germany). Volumes were filled up with water to 10 µL and incubated for 60 min at 42 °C followed an enzyme inactivation for 5 min at 90 °C. For miRNAs measurements, primers were designed manually using the software tool by Busk (Busk 2014). Primer sequences are shown in Table 2. 10 ng cDNA from cortical, hippocampal or splenic tissue were used as input for Real-Time PCR with GoTaq qPCR 2x Master Mix (Promega, Mannheim, Germany), the specific miR primers and water. SnU6RNA was used as endogenic control for all miRNAs.

**Table 2:** miRNA Primer Sequence

miR-21-5p forward primer reverse primer	5’-GCAGTAGCTTATCAGACTGATG-3’ 5’-GGTCCAGTTTTTTTTTTTTTTTCAAC-3’
miR-27a-3p forward primer reverse primer	5’-GCAGTTCACAGTGGCTAAG-3’ 5’-CCAGTTTTTTTTTTTTTTTGCGGA-3’
miR-30a-5p forward primer reverse primer	5’-GCAGTGTAAACATCCTCGAC-3’ 5’-TCCAGTTTTTTTTTTTTTTTCTTCCA-3’
miR-34a-5p forward primer reverse primer	5’-GCAGTGGCAGTGTCTTAG-3’ 5’-GGTCCAGTTTTTTTTTTTTTTTACAAC-3’
miR-204-5p forward primer reverse primer	5’-CGCAGTTCCCTTTGTCATC-3’ 5’-CCAGTTTTTTTTTTTTTTTAGGCATAG-3’
miR-Let-7a-5p forward primer reverse primer	5’-GCAGTGAGGTAGTAGGTTG-3’ 5’-GGTCCAGTTTTTTTTTTTTTTTAACTATAC-3’
snU6RNA forward primer reverse primer	5’-ATACAGAGAAGATTAGCATGGCC-3’ 5’-CGAATTTGCGTGTCATCCTTG-3’

All Real-Time PCR gene expression experiments were analysed using a QuantStudio™ 5 detection system (Thermo Fisher Scientific, Darmstadt, Germany) and the QuantStudioTM Design & Analysis Software (v1.4.3 Thermo Fisher Scientific, Darmstadt, Germany) according to the 2^-∆∆^CT method (Livak and Schmittgen 2001).

### Statistical Analyses

All experiments were performed with n = 6-8 animals per group. Sample size was calculated on markers of neuroinflammation (Paeschke et al. 2017). All data are shown as boxplots. Differences within the groups of one time point were calculated by using the non-parametric Kruskal-Wallis test and the Dunn’s multiple comparisons test as post-hoc analyses. Correlation analyses were conducted with the non-parametric Spearman correlation. A p value of < 0.05 was considered significant. All graphical and statistical analyses were performed with the software GraphPad Prism 8.0 (La Jolla, CA, USA).

## Results

### DEX Restores the LPS-Induced Inhibition of Autophagic Flux in the Spleen

As autophagy regulates immune cells (Deretic et al. 2013) and plays a crucial role in the survival of neurons (Nixon 2013), we were interested whether LPS influences the autophagic process in the spleen and brain and whether a pretreatment with DEX is able to repair possible disturbances in these organs.

**Fig. 1:**
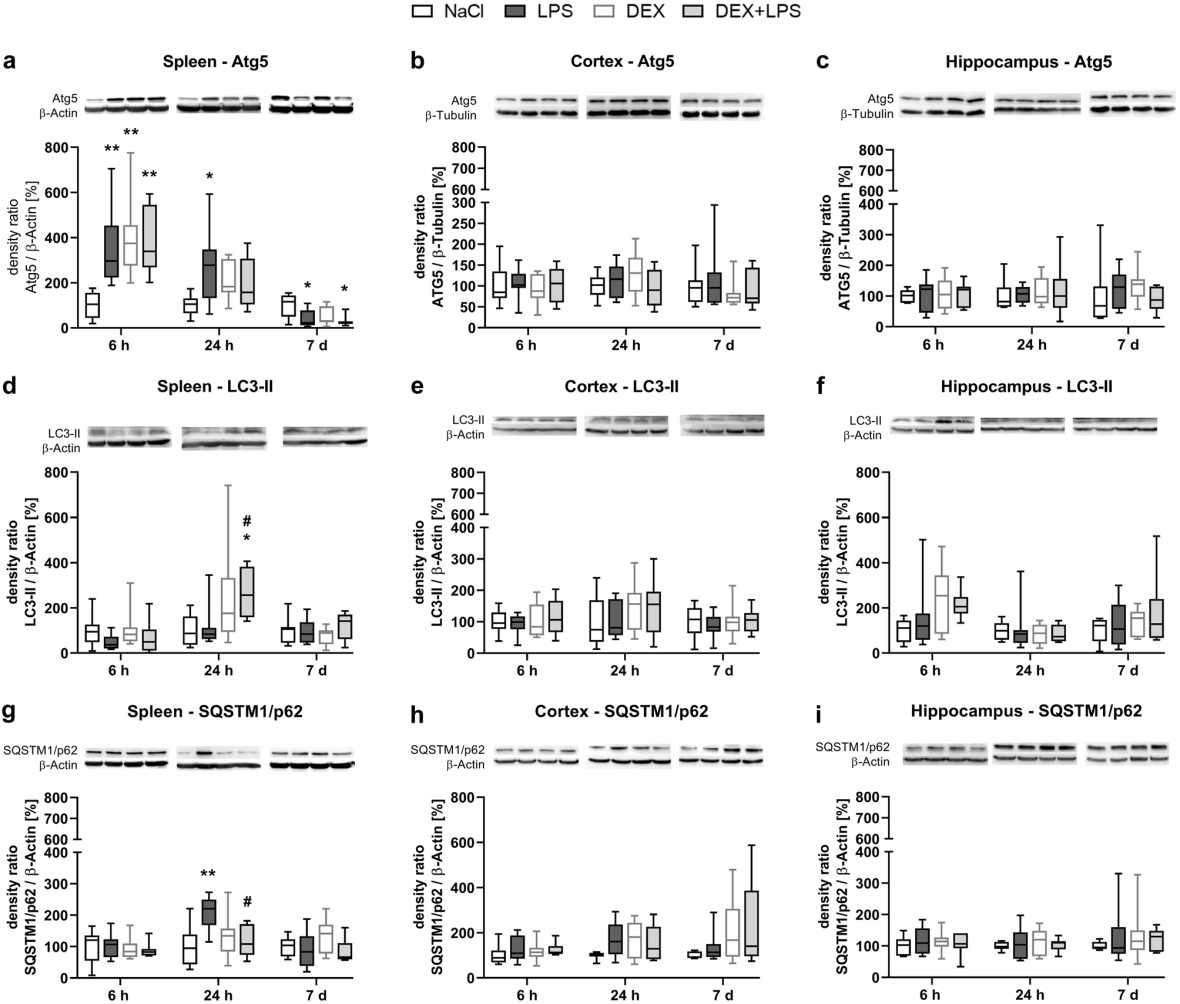
DEX restores the LPS-induced inhibition of the autophagic flux in the spleen. Changes in autophagy-associated proteins **a-c** Atg5, **d-f** LC3-II and **g-i** SQSTM1/p62 in the spleen, cortex and hippocampus were detected by immunoblotting and analysed by densitometric quantification. Results are shown as boxplots with n = 6–8 per group and data normalized to levels of NaCl-treated rats (control = 100%). **a-i** * *p* < 0.05, ** *p* < 0.01 in Kruskal–Wallis test, followed by a Dunn’s multiple comparisons post-hoc test vs NaCl group; and # *p* < 0.05 in Kruskal–Wallis test, followed by a Dunn’s multiple comparisons post-hoc test between LPS and DEX + LPS group

In the spleen, treatment with LPS increased the expression of autophagy-relevant protein Atg5 significantly after 6 h compared to the NaCl control group. DEX alone (DEX) and DEX in combination with LPS (DEX+LPS) also significantly increased Atg5 expression at this time point (Fig. 1a). After 24 h, the Atg5 expression remained upregulated in the LPS group, whereas the DEX and DEX+LPS group just showed tendencies of such an increase. In contrast, after 7 d, LPS significantly decreased the Atg5 expression, and this was also observed in the DEX+LPS, but not in the DEX group (Fig. 1a).

An intact autophagic flux, which can be detected by the amount of microtubule-associated protein 1 light chain 3 II (LC3-II) and sequestosome 1 (SQSTM1)/p62, was disturbed by LPS, but could be restored by a DEX pretreatment (DEX+LPS). LPS did not change LC3-II expression (Fig. 1d), but significantly increased the SQSTM1/p62 expression after 24 h compared to the NaCl control (Fig. 1g). In contrast, DEX+LPS increased the expression of LC3-II significantly compared to control and LPS group (Fig. 1d) but did not alter SQSTM1/p62 expression compared to controls after 24 h (Fig. 1g). Thus, we found that both, LPS and DEX, induced autophagy, but that only a pretreatment with DEX restored the LPS-induced disturbances in the autophagic flux in the spleen.

Contrary to the spleen, we did not detect any changes induced by the peripheral LPS injection, nor any effects of a DEX treatment on autophagy or the autophagic flux in the cortex (Fig. 1b, e, h) and in the hippocampus (Fig. 1c, f, i).

### DEX attenuates the LPS-induced PARP cleavage in the spleen

As autophagy and apoptosis are closely connected (Maiuri et al. 2007) and as DEX was previously described to have anti-apoptotic properties (Dardalas et al. 2019), we also examined whether DEX would change apoptosis-associated proteins in the spleen and brain.

**Fig. 2:**
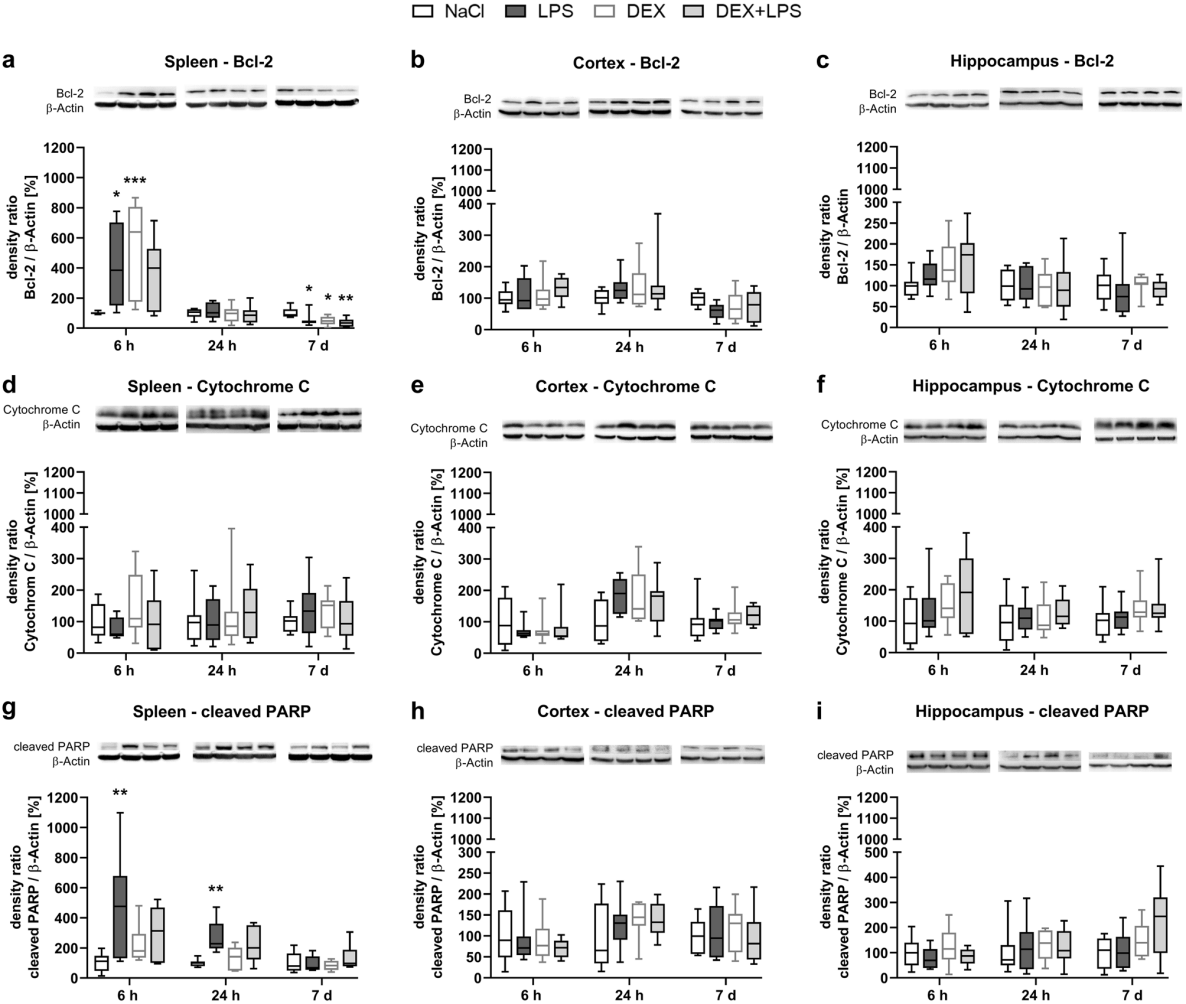
DEX attenuates PARP cleavage in the spleen. Changes in the anti-apoptotic protein **a-c** Bcl-2 and in the pro-apoptotic proteins **d-f** Cytochrome C and **g-i** cleaved PARP in the spleen and brain were detected by immunoblotting and analysed by densitometric quantification. Results are shown as boxplots with n = 6–8 per group and data normalized to levels of NaCl-treated rats (control = 100%). **a-i** * *p* < 0.05, ** *p* < 0.01, and *** *p* < 0.001 in Kruskal–Wallis test, followed by a Dunn’s multiple comparisons post-hoc test vs NaCl group

In the spleen, LPS significantly increased the expression of the anti-apoptotic protein Bcl-2 after 6 h compared to the NaCl control group. A similar change was also observed in the DEX group, whereas a DEX pretreatment (DEX+LPS) just showed tendencies of such an increase (Fig. 2a). While no differences compared to the control group were visible after 24 h anymore, LPS significantly decreased the Bcl-2 expression after 7 d, and this was also observed in the DEX and DEX+LPS group.

We also measured the release of Cytochrome C from mitochondria into the cytosol, but detected no changes in the Cytochrome C protein expression within the three treatment groups compared to control group in the spleen (Fig. 2d).

**Fig. 3:**
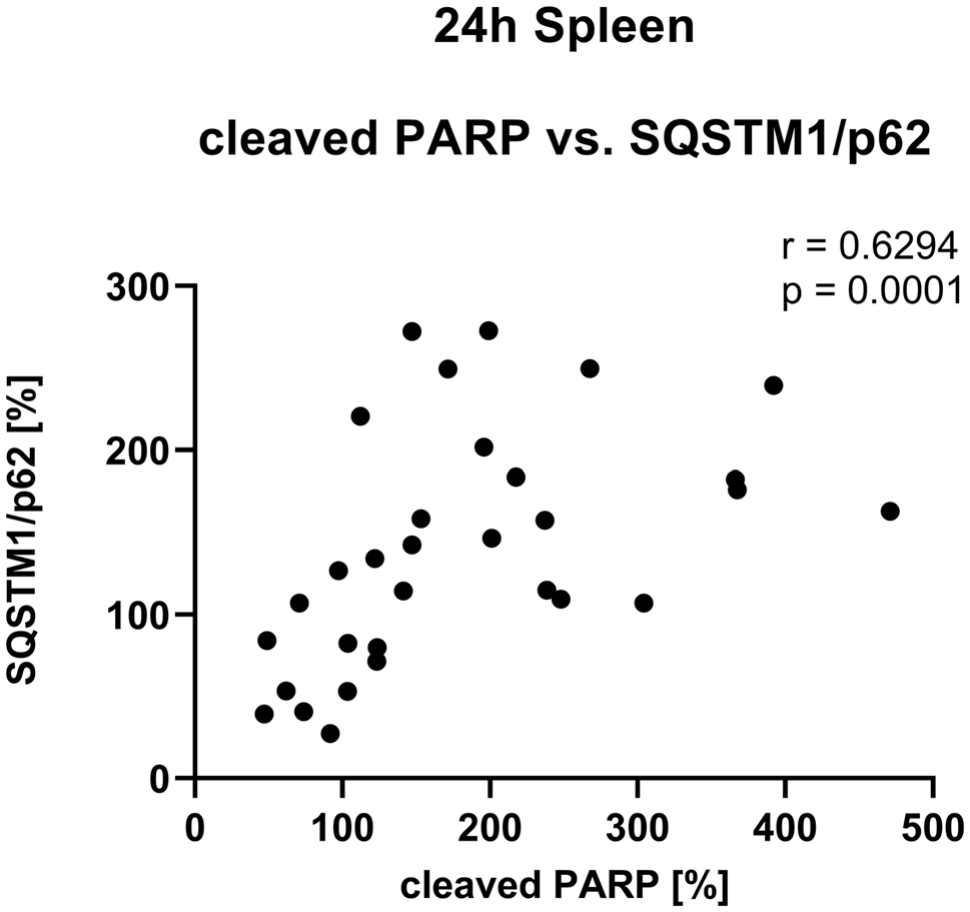
A positive monotonic correlation was found between PARP cleavage and SQSTM1/p62 after 24 h in the spleen. Results are shown as scatter plots with *n* = 31. *p* = 0.0001 between splenic cleaved PARP expression vs. SQSTM1/p62 expression after 24 h in Spearman correlation analysis with Spearman r = 0.6294

Despite the increase in Bcl-2 expression, LPS also significantly increased the protein expression of cleaved Poly (ADP-Ribose)-Polymerase (PARP) after 6 h and 24 h compared to the NaCl group (Fig. 2g). In contrast, a DEX pretreatment (DEX+LPS) did not change the cleaved PARP expression after 6 h or 24 h compared to controls, and also the DEX group showed no such changes, indicating that DEX attenuated the LPS-induced increase in cleaved PARP at both time points. In addition, we found a positive monotonic correlation between the cleaved PARP expression and SQSTM1/p62 expression after 24 h (Fig. 3), suggesting that an impaired autophagic flux might be associated with an increase in the cell death marker cleaved PARP.

In contrast to the spleen, we detected no changes in the anti-apoptotic protein Bcl-2 (Fig. 2b, c) nor in any pro-apoptotic protein (Fig. 2e-f, h−i) within the brain.

### Both, LPS and DEX, change miRNA expression in the spleen and brain

In addition, we were interested in the role of miRNAs that are known to regulate autophagy in the spleen and brain, to see whether LPS- and DEX-induced effects on autophagy were mediated by these regulatory molecules. Therefore, we measured the expression of six miRNAs, miR-21-5p, miR-27a-3p, miR-30a-5p, miR-34a-5p, miR-204-5p and miR-Let-7a-5p, all known to affect the autophagic process (Gu et al. 2020; Morgado et al. 2015; Song et al. 2015; Sun et al. 2017; Wang et al. 2014; Yan et al. 2019).

### DEX prevents the LPS-induced decrease in miR-Let-7a-5p and attenuates the decrease of miR-21-5p and miR-204-5p in the spleen

**Fig. 4:**
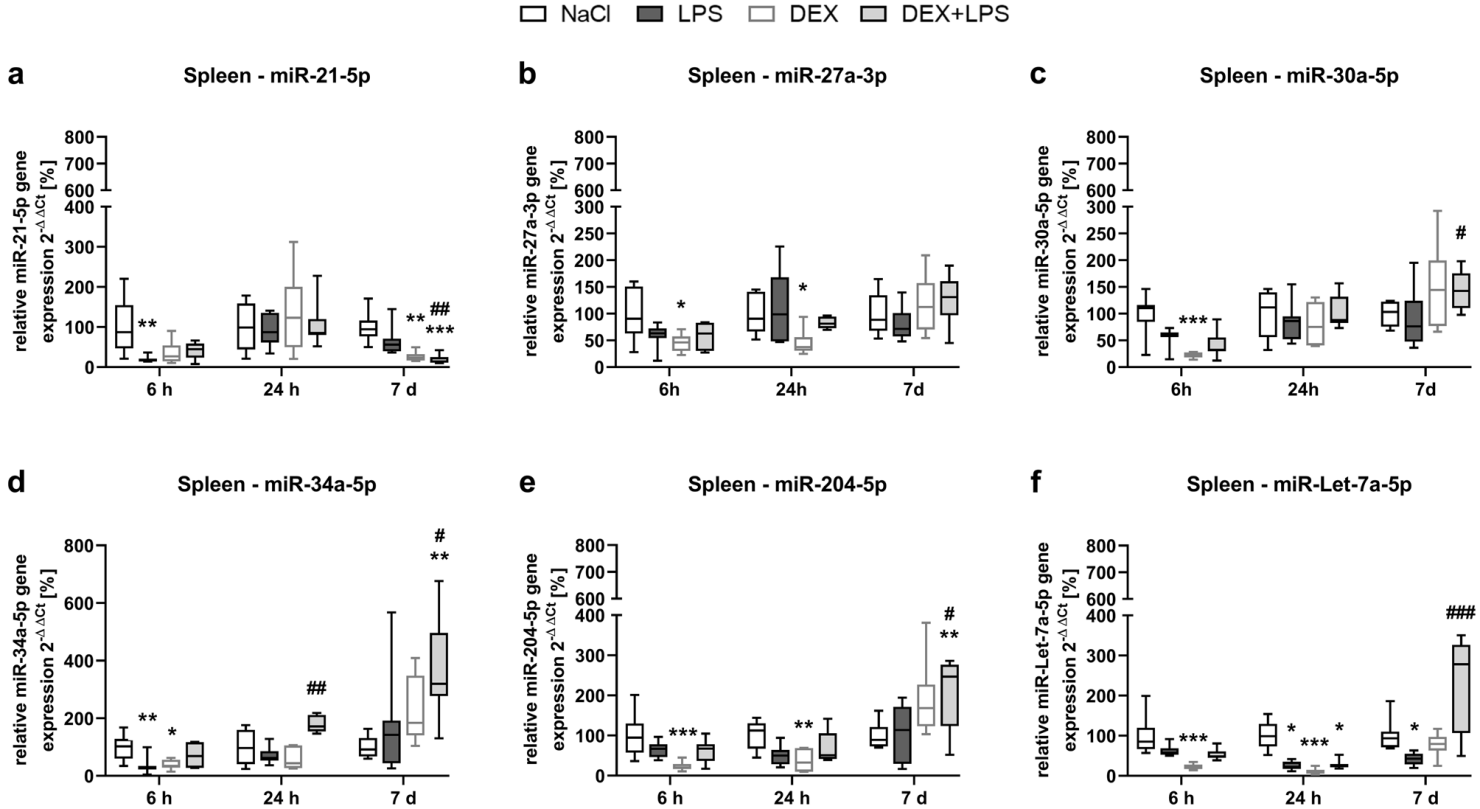
DEX prevents the LPS-induced decrease in miR-Let-7a-5p and attenuates the decrease in miR-21-5p and miR-34a-5p in the spleen. The changes in the miRNA expression of **a** miR-21-5p, **b** miR-27a-3p, **c** miR-30a-5p, **d** miR-34a-5p, **e** miR-204-5p and **f** miR-Let-7a-5p were detected by Real-Time PCR and quantified according to the 2^−∆∆^CT method (Livak and Schmittgen 2001). Results are shown as boxplots with n = 6–8 per group and data normalized to levels of NaCl-treated rats (control = 100%). a-f* *p* < 0.05, ** *p* < 0.01, and *** *p* < 0.001 in Kruskal–Wallis test, followed by a Dunn’s multiple comparisons post-hoc test vs NaCl group; and # *p* < 0.05, ## *p* < 0.01, and ### *p* < 0.001 in Kruskal–Wallis test, followed by a Dunn’s multiple comparisons post-hoc test between LPS and DEX + LPS group

LPS downregulated the expression of three out of six studied miRNAs in the spleen (Fig. 4a-f): it significantly downregulated miR-21-5p (Fig. 4a) and miR-34a-5p expression after 6 h (Fig. 4d) and miR-Let-7a-5p after 24 h and 7 d compared to NaCl control group (Fig. 4f). In contrast, a pretreatment with DEX (DEX+LPS) did not alter the expression of these miRNAs compared to control and, in the case of miR-Let-7a-5p, even significantly increased miRNA expression compared to LPS after 7 d, indicating that DEX+LPS attenuated or prevented the LPS-induced changes in miRNA expression. Moreover, both, DEX and DEX+LPS, significantly increased and/or decreased the expression of all six investigated miRNAs at different time points: after 6 h, DEX reduced the expression of all miRNAs, except for miR-21-5p compared to control group; after 24 h, DEX reduced the expression of miR-27a-5p, miR-204-5p, and miR-Let-7a-5p compared to controls and DEX+LPS increased the expression of miR-34a-5p compared to the LPS; after 7 d, DEX reduced the expression of miR-21-5p compared to controls and, in addition, DEX+LPS also reduced the expression of miR-21-5p as well as increased the expression of miR-30a-5p, miR-34a-5p, miR-204-5p, and miR-Let-7a-5p compared to LPS and, in the case of miR-34a-5p and miR-204-5p also compared to the control group at this time point.

**Fig. 5:**
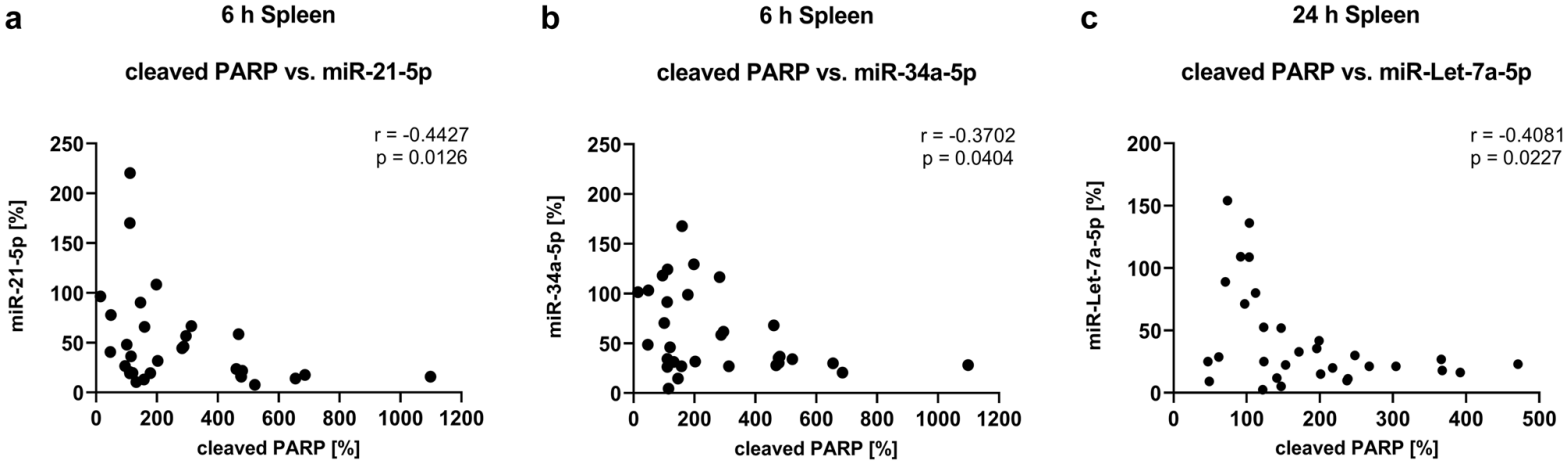
A negative monotonic correlation was found between PARP cleavage and miR-21-5p and miR-34a-5p after 6 h and miR-Let-7a-5p after 24 h in the spleen. Results are shown as scatter plots with n = 31. *p* < 0.05 between splenic cleaved PARP expression vs. **a** miR-21-5p and **b** miR-34a-5p expression after 6 h and **c** miR-Let-7a-5p expression after 24 h in Spearman correlation analysis with Spearman r = -0.4427, -0.3702 and -0.4081 respectively

Furthermore, we conducted correlation analyses, as we were interested whether the changes seen on miRNA level correlated with the changes in autophagy and apoptosis-associated proteins in the spleen. While we found no correlations between miRNAs and autophagy-associated proteins, we found negative monotonic correlations between cleaved PARP and three miRNAs. Cleaved PARP correlated with both, miR-21-5p and miR-34a-5p, after 6 h (Fig. 5a, b), and with miR-Let-7a-5p after 24 h (Fig. 5c).

### DEX does not attenuate the LPS-induced changes in miRNA expression in the cortex

**Fig. 6:**
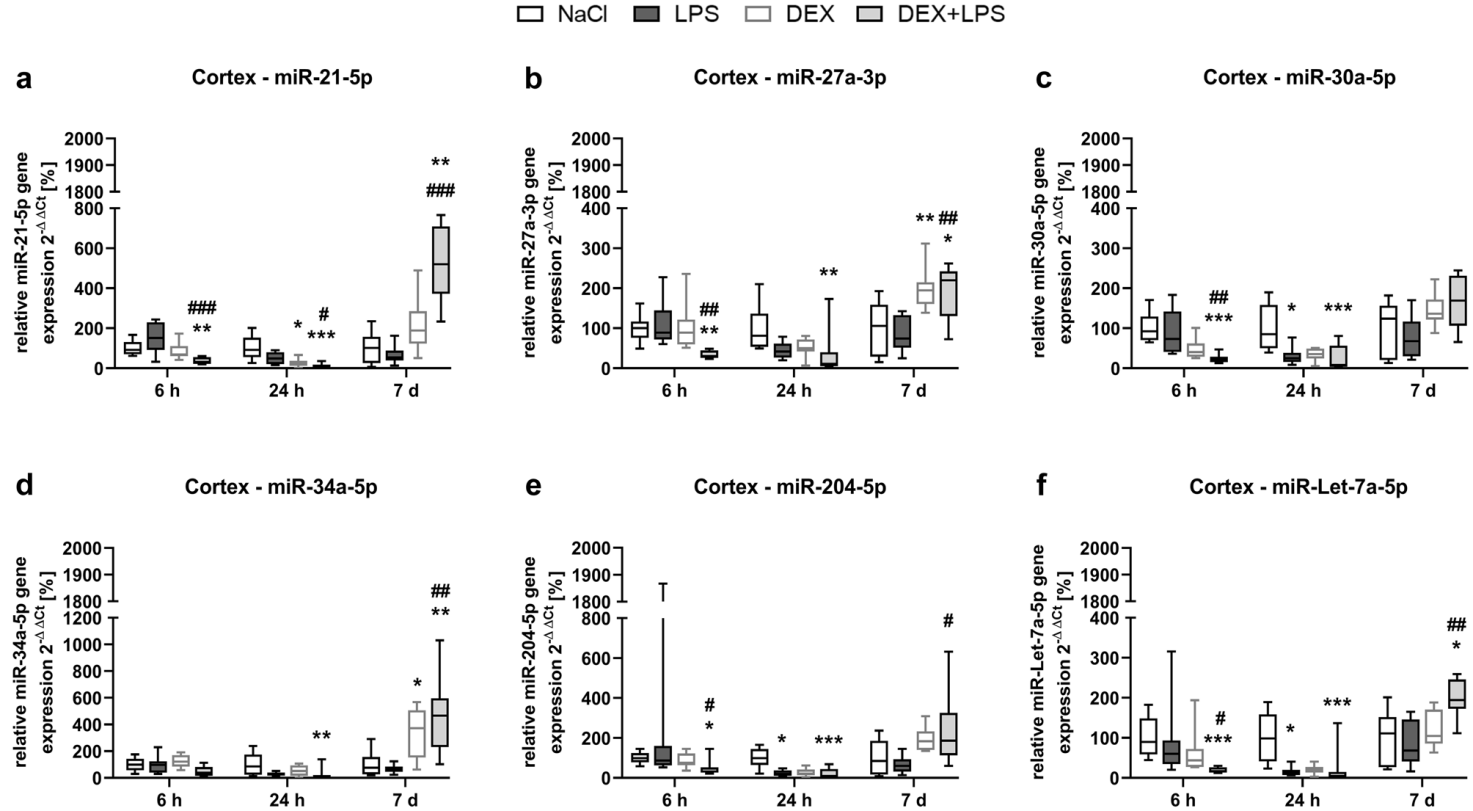
DEX does not attenuate the LPS-induced changes in miR-30a-5p, miR-204-5p and miR-Let-7a-5p expression in the cortex. The changes in the miRNA expression of **a** miR-21-5p, **b** miR-27a-3p, **c** miR-30a-5p, **d** miR-34a-5p, **e** miR-204-5p and **f** miR-Let-7a-5p were detected by Real-Time PCR and quantified according to the 2^−∆∆^CT method (Livak and Schmittgen 2001). Results are shown as boxplots with *n* = 6–8 per group and data normalized to levels of NaCl-treated rats (control = 100%). **a-f** * *p* < 0.05, ** *p* < 0.01, and *** *p* < 0.001 in Kruskal–Wallis test, followed by a Dunn’s multiple comparisons post-hoc test vs NaCl group; and # *p* < 0.05, ## *p* < 0.01, and ### *p* < 0.001 in Kruskal–Wallis test, followed by a Dunn’s multiple comparisons post-hoc test between LPS and DEX + LPS group

In the cortex, LPS also decreased the expression of three out of the six studied miRNAs (Fig. 6a-f): it significantly reduced the expression of miR-30a-5p (Fig. 6c), miR-204-5p (Fig. 6e), and miR-Let-7a-5p (Fig. 6f) after 24 h compared to the NaCl control group. In contrast to the spleen, a pretreatment with DEX (DEX+LPS) also reduced the expression of these three miRNAs significantly, indicating that DEX was unable to prevent the LPS-induced downregulations. Similar to the spleen, we found that both, DEX and DEX+LPS, significantly increased and/or decreased the expression of all investigated miRNAs at different time points: after 6 h, DEX+LPS reduced the expression of all miRNAs except for miR-34a-5p compared to the control and LPS group; after 24 h, DEX reduced the expression of miR-21-5p compared to the control group, while DEX+LPS downregulated miR-21-5p compared to control and LPS group, and also reduced the expression of all other miRNAs compared to control group; and after 7 d, DEX increased the expression of miR-27a-5p and miR-34a-5p compared to controls, while DEX+LPS increased the expression of miR-21-5p, miR-27a-5p, miR-34a-5p and miR-Let-7a-5p compared to control and LPS group, and, in the case of miR-204-5p, compared LPS group. Notably, in the cortex, DEX+LPS induced more significant changes to the miRNA expression than DEX and, similar to the spleen, DEX+LPS often induced a significantly different miRNA expression compared to the LPS group.

### DEX prevents LPS-induced increase in miR-30a-5p and attenuates decrease of miR-21-5p and miR-204-5p in the hippocampus

**Fig. 7:**
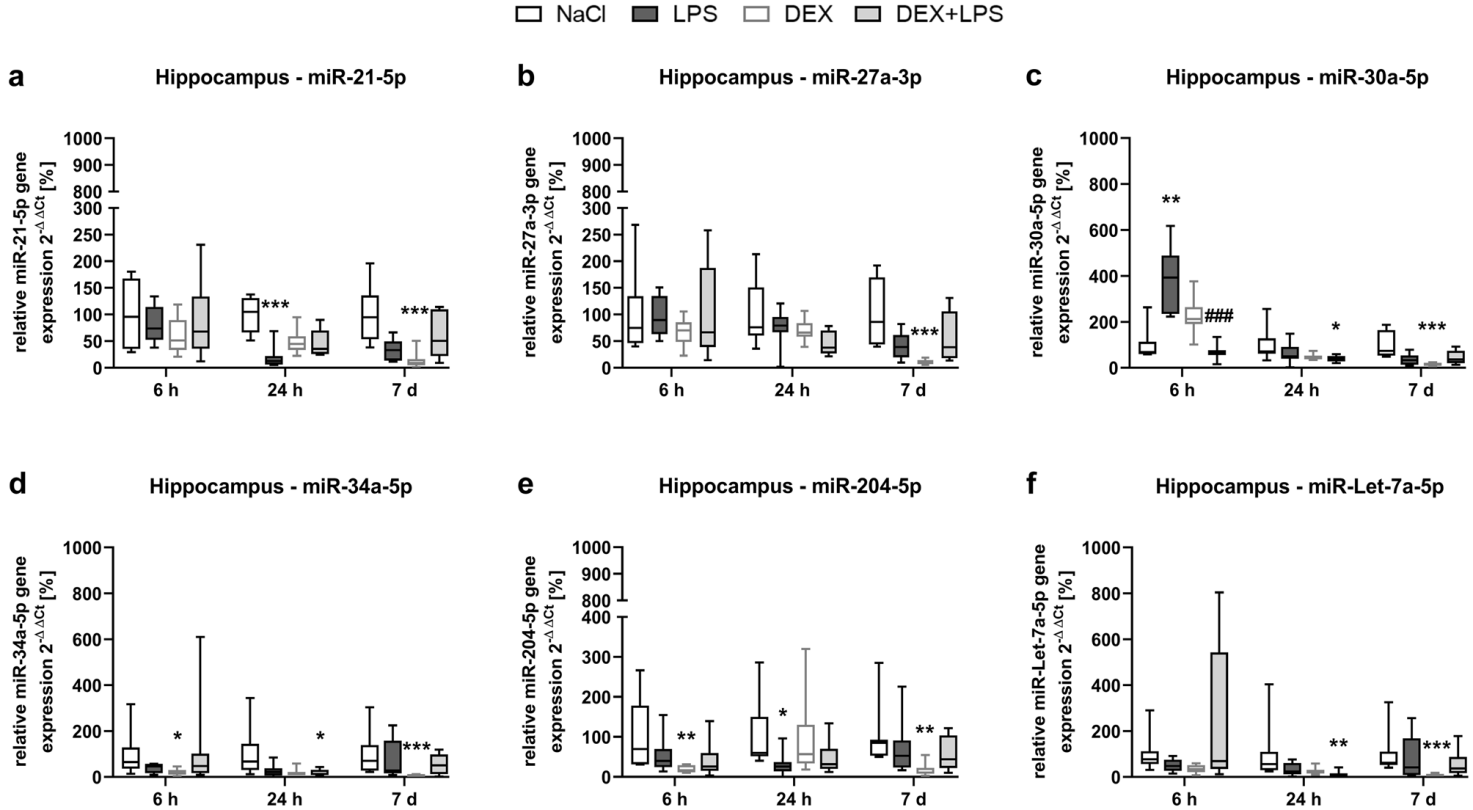
DEX prevents the LPS-induced increase in miR-30a-5p and attenuates the decrease in miR-21-5p and miR-204-5p in the hippocampus. The changes in the miRNA expression of **a** miR-21-5p, **b** miR-27a-3p, **c** miR-30a-5p, **d** miR-34a-5p, **e** miR-204-5p and **f** miR-Let-7a-5p were detected by Real-Time PCR and quantified according to the 2^−∆∆^CT method (Livak and Schmittgen 2001). Results are shown as boxplots with n = 6–8 per group and data normalized to levels of NaCl-treated rats (control = 100%). **a-f** * *p* < 0.05, ** *p* < 0.01, and *** *p* < 0.001 in Kruskal–Wallis test, followed by a Dunn’s multiple comparisons post-hoc test vs NaCl group; and ### *p* < 0.001 in Kruskal–Wallis test, followed by a Dunn’s multiple comparisons post-hoc test between LPS and DEX + LPS group

Again, in the hippocampus, LPS up- as well as downregulated the expression of half of the investigated miRNAs (Fig. 7a–f): it increased the expression of miR-30a-5p after 6 h (Fig. 7c) and decreased the expression of miR-21-5p (Fig. 7a) as well as miR-204-5p (Fig. 7e) after 24 h compared to the NaCl control group. Similar to the spleen and contrary to the cortex, a DEX pretreatment (DEX+LPS) did not alter the expression of miR-21-5p and miR-204-5p after 24 h compared to controls and successfully prevented the LPS-induced increase in miR-30a-5p expression, indicating that DEX+LPS prevented or attenuated the LPS-induced changes in miRNA expression. Similar to the spleen and the cortex, we found that both, DEX and DEX+LPS, significantly increased and/or decreased all investigated miRNAs at different time points: after 6 h, DEX reduced the expression of miR-34a-5p and miR-204-5p compared to control group, while after 24 h, DEX+LPS reduced the expression of miR-30a-5p, miR-34a-5p and miR-Let-7a-5p compared to control group, and after 7 d, DEX reduced the expression of all six investigated miRNAs compared to control group. Contrary to the cortex, DEX modified the miRNA expression more profoundly than LPS or DEX+LPS and there were hardly any significant differences between the DEX+LPS and LPS group in the hippocampus. Furthermore, only within this brain region, LPS was able to upregulate miRNA expression (Fig. 7c).

### Both, DEX and LPS, change the expression of acetylcholine and the activity of acetylcholinesterase in the spleen and brain

Recent studies indicate that an association between the cholinergic anti-inflammatory pathway and the DEX-mediated neuro- and organo-protection might exist (Ding et al. 2019; Zhu et al. 2016). Thus, we tested whether LPS changed molecules of the cholinergic system and whether a DEX pretreatment was able to influence these changes.

**Fig. 8:**
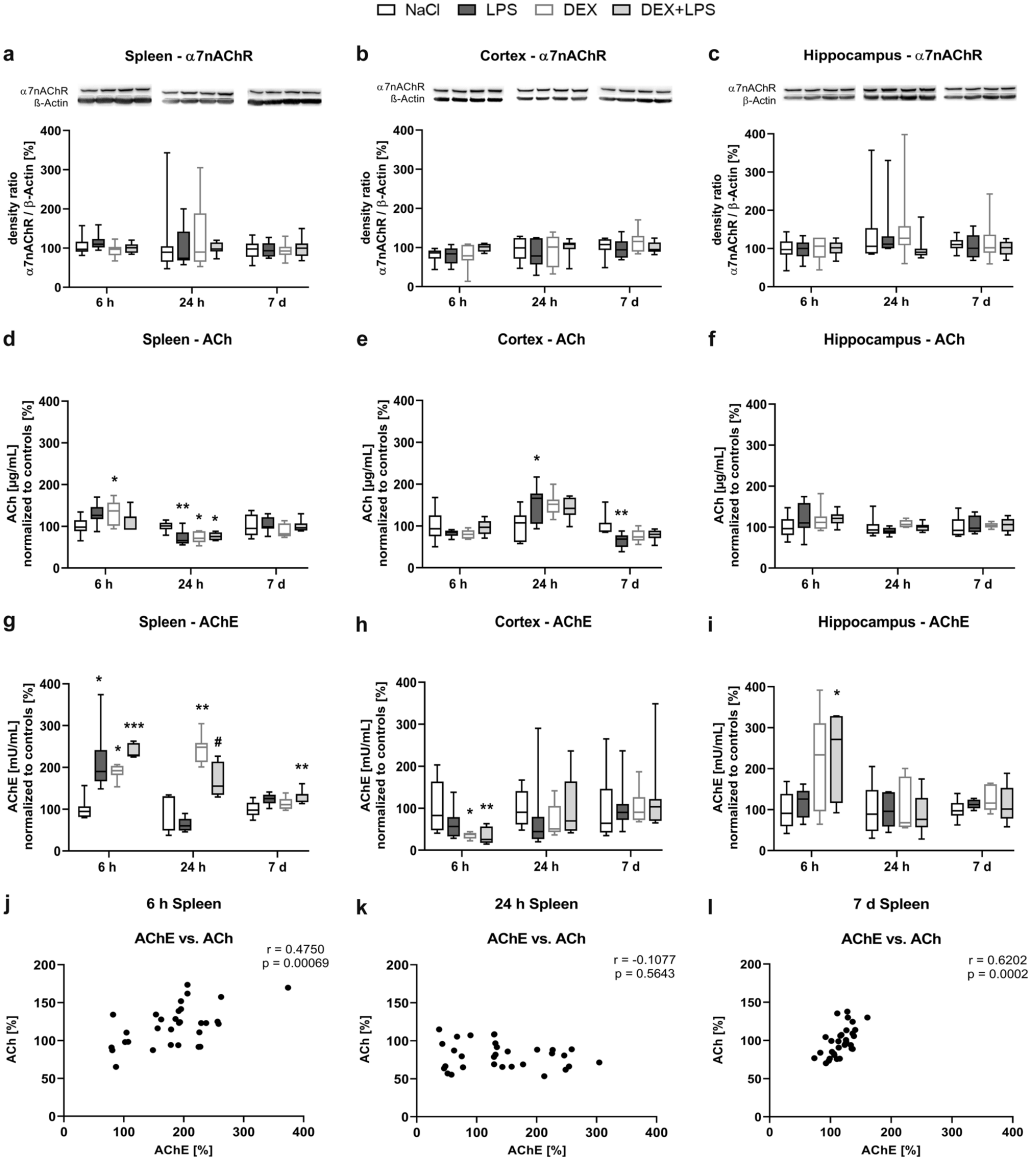
Both, DEX and LPS change the expression of acetylcholine and the activity of acetylcholinesterase in the spleen and brain. **a-c** Protein expression of α7nAChR was detected by immunoblotting and densitometric quantification. **d-f** Acetylcholine level and **g-i** acetylcholinesterase activity were measured with the Amplex® Red Acetylcholine/Acetylcholinesterase Assay. Results are shown as boxplots with *n* = 6–8 per group and data normalized to levels of NaCl-treated rats (control = 100%). **a-i** * *p* < 0.05, ** *p* < 0.01, and *** *p* < 0.001 in Kruskal–Wallis test, followed by a Dunn’s multiple comparisons post-hoc test vs NaCl group; and # p < 0.05 in Kruskal–Wallis test, followed by a Dunn’s multiple comparisons post-hoc test between LPS and DEX + LPS group. **j-l** correlation analyses of acetylcholinesterase and acetylcholine showing a positive monotonic correlation after 6 h and 7 d, but not after 24 h in the spleen. Results are shown as scatter plots with *n* = 31–32. *p* < 0.001 between acetylcholinesterase activity vs. acetylcholine expression after **j** 6 h, *p* > 0.05; after **k** 24 h *p *> 0.05; and after **l** 7 d *p* < 0.001 in Spearman correlation analysis with Spearman r = 0.4750, -0.1077 and 0.6202 respectively

We detected no alterations in the protein expression of α7nAChR, neither in the spleen (Fig. 8a), nor in the brain (Fig. 8b, c), but observed changes in ACh expression (Fig. 8d-f) and AChE activity (Fig. 8g-i) in both organs.

In the spleen, the expression of ACh remained at control level in the LPS and DEX+LPS group after 6 h, but DEX significantly increased ACh expression (Fig. 8d). After 24 h, LPS significantly decreased the expression of ACh below control levels, and also DEX and DEX+LPS induced a similar decrease (Fig. 8d). No changes compared to controls were found after 7 d. In contrast to ACh, we detected more pronounced effects of both, LPS and DEX, regarding the AChE activity. In the spleen, LPS significantly increased the AChE activity after 6 h, and also DEX and DEX+LPS upregulated the AChE activity to a similar level compared to controls (Fig. 8g). After 24 h, the increased AChE activity returned to control level in the LPS group. In contrast, AChE activity remained significantly increased in the DEX and DEX+LPS group compared to control and LPS group respectively, indicating that DEX prevented the LPS-induced decrease in AChE activity. After 7 d, AChE activity remained upregulated only in the DEX+LPS group. When correlating AChE and ACh, we found a positive monotonic correlation after 6 h and 7 d (Fig. 8j, l), but not after 24 h (Fig. 8k), which further underlines the AChE activating effect of DEX at this time point.

In the brain, ACh expression changed within the cortex (Fig. 8e), but not within the hippocampus (Fig. 8f). In the cortex, LPS significantly increased the ACh expression after 24 h and decreased it after 7 d compared to controls, whereas DEX and DEX+LPS only showed tendencies of such an increase and decrease respectively. In contrast to the spleen, LPS did not modify the AChE activity in the brain, but only DEX did (Fig. 8h, i). After 6 h, both, DEX and DEX+LPS significantly reduced AChE activity in the cortex compared to control group (Fig. 8h), whereas in the hippocampus the opposite effect was observed, as DEX+LPS significantly increased AChE activity after 6 h (Fig. 8i).

### Discussion

This study was conducted to understand the mechanisms behind the DEX-mediated neuro- and organo-protection. We have previously shown that LPS treatment increased the pro-inflammatory cytokines Interleukin-1β (IL-1β) and Tumor necrosis factor-α (TNF-α) as well as upregulated miR-124, miR-132, miR-134 and miR-155 in the hippocampus and cortex, and that DEX prevented this (Paeschke et al. 2017). In the present study, we have now added new insights into the DEX-mediated protection during inflammation by investigating its role in autophagy and cell death as well as its effect on associated miRNAs and the cholinergic system in the spleen and brain.

Neither LPS, nor DEX induced changes in autophagy and apoptosis-associated proteins in the cortex or hippocampus. The missing effect in the brain might indicate that the LPS injection alone was probably not sufficient to actually induce apoptosis, but only to increase pro-inflammatory cytokines, as previously shown (Paeschke et al. 2017). Similar results were observed earlier (Kalb et al. 2013) and thus indicate that a combination of damage-associated molecular patterns (DAMPs) and pathogen-associated molecular patterns (PAMPs) may be necessary to strongly impact the brain.

In contrast, both DEX and LPS, had distinctively different effects on such proteins in the spleen. While early on all three treatment options (LPS, DEX and DEX+LPS) increased the expression of the autophagy protein Atg5, which is involved in the elongation process (Mizushima et al. 2011), only in the presence of LPS Atg5 was altered at later time points, indicating that LPS might have had a long-lasting effect on autophagosome formation. However, LPS also impaired the autophagic flux, as indicated by measuring LC3-II and SQSTM1/p62, whereas DEX was able to restore the autophagic flux in the spleen.

The spleen, as the largest secondary lymphoid organ in the body, hosts a wide range of immunological functions (Lewis et al. 2019) and autophagy in turn holds a crucial role within immune cells (Deretic et al. 2013). Murine studies showed that defects in autophagy in the presence of LPS caused an increase in pro-inflammatory cytokines and a higher mortality, whereas pharmacological activation of autophagy reversed both (Harris 2011; Jones et al. 2013). Our results are in line with findings showing that DEX protected the kidney from LPS induced acute kidney injury by upregulating autophagy (Zhao et al. 2020).

Moreover, the apoptosis of immune cells in immune relevant organs is a major hallmark of sepsis pathophysiology and approaches aimed to prevent this are promising therapeutic options (Luan et al. 2015). While both, LPS and DEX, increased and decreased the expression of the anti-apoptotic protein Bcl-2 depending on the time point, only LPS also increased PARP cleavage. In contrast, the DEX+LPS-treated animals attenuated PARP cleavage, indicating a possible anti-apoptotic effect of DEX in spleen. Furthermore, as cleaved PARP and SQSTM1/p62 correlated positively in the spleen, our results might point towards the importance of a proper functioning autophagy in immune cells. Thus, the autophagy restoring effect of DEX holds a promising approach to prevent organ damage. As we did not find any changes on Cytochrome C protein levels, we assumed that the here suspected mechanism was mitochondria-independent. Anti-apoptotic effects of DEX in the spleen were also described by others (Qiao et al. 2009) and were observed in other organs, too (Rong et al. 2017; Zhao et al. 2020).

MicroRNAs are key regulators of autophagy, apoptosis and inflammation (Gaudet et al. 2018; Su et al. 2015). In our study, we investigated six miRNAs, which are all known to regulate autophagy and inflammation or apoptosis, with miR-21-5p, miR-27a-3p, miR-30a-5p, miR-34a-5p, and miR-204-5p inhibiting various steps of autophagy (Gu et al. 2020; Morgado et al. 2015; Sun et al. 2017; Wang et al. 2014; Yan et al. 2019), and miR-Let-7a and its family members activating this process (Song et al. 2015).

In this study, we found organ-specific effects of LPS on miRNA expression, which DEX was able to prevent or attenuate in the spleen and hippocampus. Although we observed no effects of the differentially regulated miRNAs on autophagy-associated proteins, we detected a negative correlation between the LPS-altered miRNAs in the spleen (miR-21-5p, miR-34a-5p and miR-Let-7a-5p) and the cell death marker cleaved PARP, indicating that these miRNAs might have rather affected inflammation and cell death mechanisms than autophagy in our model.

Along with miR-21, miR-27a, miR-34a, and miR-Let-7a have been described to have anti-inflammatory properties (Gaudet et al. 2018; Jian et al. 2020; Sheedy 2015; Stachurska et al. 2014), whereas miR-30a-5p and miR-204-5p are reported to exert a pro-inflammatory and/or -apoptotic effect (Wang et al. 2014; Yan et al. 2019). miR-21 is a key modulator of inflammation, which is upregulated in activated immune cells to promote the resolution of inflammation (Sheedy 2015) and to protect the CNS from apoptosis (Gaudet et al. 2018). miR-27a is reported to downregulate pro-inflammatory cytokines (Stachurska et al. 2014) and apoptosis (Sabirzhanov et al. 2014). miR-34a also limits an excessive pro-inflammatory response by targeting Notch1 (Jian et al. 2020) and reducing various pro-inflammatory cytokines (Jian et al. 2020), but was also shown to have detrimental effects in the CNS (Chua and Tang 2019). miR-Let-7a and its family members are upregulated in the plasma during the resolution of inflammation (Silva et al. 2018). They provide a negative feedback to limit inflammation (Gaudet et al. 2018) by reducing the expression of pro-inflammatory cytokines and apoptotic proteins (Gaudet et al. 2018).

Thus, the negative correlations we found in the spleen, indicate that LPS might have mediated its detrimental effects by inhibiting the expression of these anti-inflammatory miRNAs, whereas DEX might have attenuated this effect of LPS, as it neither changed the expression of these miRNAs, nor increased PARP cleavage significantly. Furthermore, the effect of DEX on miRNA expression extended to the brain as well, as it prevented the LPS-mediated increase of the pro-inflammatory miR-30a-5p and attenuated the decrease in miR-21-5p and miR-204-5p in the hippocampus.

Moreover, while LPS mainly downregulated miRNA expression, DEX frequently also upregulated miRNA expression at later time points. Similar modulation patterns were also observed elsewhere: In murine whole blood samples, in which miRNAs were rather down- than upregulated 6 h after an LPS injection (Hsieh et al. 2012) and in a bone injury model, in which miRNA expressions mainly decreased within the acute phase of an inflammation, but increased within the resolving phase (Silva et al. 2018). In light of these findings, our results might point toward an inflammation resolving effect of DEX by promoting miRNA expression.

Interestingly, the effects of DEX on miRNA expression were visible up to 7 days after treatment, demonstrating a long-lasting miRNA modulation, which might have extended the effects of the drug itself, as – with an elimination half-life of about 2 hours – DEX is metabolized relatively fast (Nguyen et al. 2017).

It is also worth noting that we observed DEX-mediated patterns in miRNA expression in all three tissues, indicating that a DEX administration might have altered common upstream factors of these miRNAs. However, as we did not detect such patterns previously (Paeschke et al. 2017), this observation needs further investigations.

The cholinergic anti-inflammatory pathway is a major communication route between the CNS and the immune system. It modulates the immune response through neural inhibition via the activation of the vagus nerve and the release of ACh, which activates the α7nAChR located on macrophages and other immune cells and in turn regulates the cytokine release (Pavlov et al. 2003). Recent studies indicate that a blockage of the α7nAChR or a vagotomy vanishes the effects of DEX (Zhu et al. 2016). Therefore, we were interested how inflammation and a prevention with DEX alter the expression of α7nAChR and ACh as well as the activity of the ACh limiting enzyme AChE.

Both, LPS and DEX, clearly influenced ACh and AChE, but neither changed α7nAChR expression in any tissue. This is in contrast to other findings, showing an increased expression of this receptor upon DEX treatment (Rong et al. 2017). However, the modulation of α7nAChR upon an inflammatory stimulus is still controversial, as others also reported a decrease in α7nAChR expression (Hoover et al. 2020; Lykhmus et al. 2016).

Regardless of the treatment option, the changes in the ACh expression appeared rather similar, whereas the changes in the AChE activity were almost exclusively attributable to DEX in both organs. Although both, LPS and DEX, initially increased the AChE activity in the spleen, only the presence of DEX induced an enhanced AChE activity after 24 h, which was further emphasized by the missing resonance between ACh and AChE at this time point. Interestingly, in the brain, we found a region-specific opposite effect of DEX on AChE activity, which we need to investigate further. We could not find any reports on how DEX affects AChE activity, but only on how an inflammatory stimulus, such as LPS, affects this enzyme - by decreasing its activity and gene expression in the spleen (Hoover et al. 2020) and cortex (Lykhmus et al. 2016). As AChE is generally seen as a detrimental factor due to its ACh-hydrolysing function, this was interpreted as a support mechanism to strengthen the cholinergic anti-inflammatory pathway. However, in our study, we observed no indications that an increased AChE activity has detrimental effect, as this neither led to a reduced ACh expression, nor to an increase in apoptosis markers. In fact, AChE has been recognized to have diverse roles outside the cholinergic system (Soreq and Seidman 2001). AChE is described to play a role in neurite outgrowth and neuromuscular junction development and has important functions during haematopoiesis, where it regulates the growth of erythrocytes and platelets (Soreq and Seidman 2001). Studies investigating the effect of AChE expression on clinical outcome found that surgery or inflammation induced low serum cholinesterase levels (Kamolz et al. 2002; Muller et al. 2019), which were partially associated with a higher mortality (Kamolz et al. 2002). This suggests that an increased AChE activity might have added to the organo-protective effects of DEX. Furthermore, as α7nAChR is also activated by choline (Alkondon et al. 1997), it should be considered that an increased AChE activity cannot be regarded as a fully negative regulator of the cholinergic system.

There are several limitations to our study. As we found no direct association between miRNAs and the autophagy-associated proteins, our results emphasize that miRNA-mediated protein regulation is a highly complex process, in which many influencing factors have to be taken into account: 1) one single miRNA is able to regulate up to 100 different target genes and vice versa (Mukherji et al. 2011); 2) the effects of miRNAs on protein output are highly dependent on the miRNA and target mRNA levels within a given tissue (Baek et al. 2008); and 3) miRNA-mediated protein repression is rather modest and thus, such regulations should be considered a fine-tuning rather than an on-off-switch of gene expression (Mukherji et al. 2011). As there are only a few studies on the influence of DEX on these miRNAs (Ha Sen Ta et al. 2019; Xing et al. 2020), more are needed to address how the DEX-induced changes in miRNA expression affect the organism. Furthermore, as we looked at the effects of LPS and DEX on the whole spleen, we still need to clarify the effect of DEX on the type of (immune) cell. It was previously reported that, although each leukocyte subpopulation is generally able to exhibit a cholinergic phenotype, mainly monocytes/macrophages increased theirs during sepsis and that AChE is mainly located within large cells of the red pulp, indicating that this enzyme is not located on leukocytes, but rather on megakaryocytes (Hoover et al. 2020). As AChE also holds functions outside the cholinergic system, future studies are also needed to understand how DEX affects the other roles of AChE. In addition, we measured the change in expression of the α7nAChR, but not its activation status. Thus, it is unclear if α7nAChR was involved in the DEX-mediated effects. Also, the different methodical approaches among studies must be considered. We might have missed the time point at which α7nAChR expression was modified by our treatment, as some recorded a decrease in the α7nAChR level after 8 h in the spleen (Hoover et al. 2020), while others found a change of this receptor after 3 days in the brain (Lykhmus et al. 2016). Furthermore, as our earliest observation time point was 6 h, we might have missed the early effects on the immune system induced by LPS and DEX. Other investigations have pointed out that DEX is able to prevent the LPS-induced increase of the pro-inflammatory cytokines TNF-α, IL-6 and IL-1ß as early as 2 h post injection in the plasma and spleen of mice (Liu et al. 2015). A similar early preventive effect of DEX on the induction of pro-inflammatory cytokines was also observed in the plasma of LPS-treated rats (Taniguchi et al. 2008). However, the different doses and application routes must also be taken into account when comparing such studies. While we used a relatively mild LPS dose (1 mg/kg body weight) and moderate dose of DEX (5 µg/kg bodyweight), other studies observed those effects under much higher concentrations of LPS and DEX respectively or under a different administration method (e.g. intravenous infusion). Furthermore, as we have previously shown in the same model that the pro-inflammatory cytokine production within the brain was prevented by DEX only after 24 h but not after 6 h, this might indicate that under our conditions the preventive effect of DEX might have needed more time to fully develop. Nonetheless, these effects were observed in the brain, but not the plasma or spleen and thus we cannot draw any conclusions on the effect of DEX in the periphery. Therefore, the early effects of DEX on miRNA expression, autophagy and the cholinergic anti-inflammatory reflex might have been mediated through the rapid effect of DEX on cytokine production within the blood and spleen as indicated by other studies (Liu et al. 2015; Taniguchi et al. 2008). Moreover, as we investigated an α2-AR agonist and used isoflurane as an anaesthetic, the additional effect of isoflurane must also be taken into account, as volatile anaesthetics have been reported to alter the function of G protein coupled receptors (GPCRs), such as the α2-AR (Ishizawa 2007). It was shown that the effect of isoflurane anaesthesia was prolonged when G protein signalling was inhibited, indicating a direct action of isoflurane on GPCRs (Icaza et al. 2009) and that isoflurane anaesthesia resulted in the inhibition of the high-affinity state of several GPCRs including the α2-AR (Seeman and Kapur 2003). As a preference between ligands and high-affinity states of GPCRs exists (Shalgunov et al. 2019), isoflurane might have weakened the binding of DEX to the α2-AR by inhibiting the high-affinity state of this GPCR. Lastly, as we only used male rats, sex differences regarding the immune response must also be taken into account when comparing studies (Spychala et al. 2017).

All in all, the herein reported results underline the complex mode of action DEX exerts within an organism after a systemic inflammation. DEX restored the LPS-induced impairments in the autophagic flux in the spleen, prevented and attenuated certain LPS-induced miRNA modifications in the spleen and hippocampus and altered molecules of the cholinergic anti-inflammatory reflex in both, the spleen and brain. In addition, DEX had a general modulating effect on miRNAs associated with autophagy and inflammation, further highlighting the different molecular pathways that DEX modifies during LPS-induced inflammation. Our results add new insights into the working mechanisms of DEX and provide new perspectives for future research on the protective mechanisms of this α2-AR agonist.

## Data Availability

Any raw data related to this manuscript may be requested from the corresponding author.
